# Predictive Biomarkers for a Personalized Approach in Resectable Pancreatic Cancer

**DOI:** 10.3389/fsurg.2022.866173

**Published:** 2022-05-04

**Authors:** Valeria Merz, Domenico Mangiameli, Camilla Zecchetto, Alberto Quinzii, Silvia Pietrobono, Carlo Messina, Simona Casalino, Marina Gaule, Camilla Pesoni, Pasquale Vitale, Chiara Trentin, Michela Frisinghelli, Orazio Caffo, Davide Melisi

**Affiliations:** ^1^Medical Oncology Unit, Santa Chiara Hospital, Trento, Italy; ^2^Digestive Molecular Clinical Oncology Research Unit, Università degli Studi di Verona, Verona, Italy; ^3^Investigational Cancer Therapeutics Clinical Unit, Azienda Ospedaliera Universitaria Integrata, Verona, Italy; ^4^Oncology Unit, A.R.N.A.S. Civico, Palermo, Italy

**Keywords:** resectable pancreatic cancer, predictive factors, neoadjuvant therapy, preoperative treatment, target therapy, molecular profiling, tumor microenvironment, microbiota

## Abstract

The mainstay treatment for patients with immediate resectable pancreatic cancer remains upfront surgery, which represents the only potentially curative strategy. Nevertheless, the majority of patients surgically resected for pancreatic cancer experiences disease relapse, even when a combination adjuvant therapy is offered. Therefore, aiming at improving disease free survival and overall survival of these patients, there is an increasing interest in evaluating the activity and efficacy of neoadjuvant and perioperative treatments. In this view, it is of utmost importance to find biomarkers able to select patients who may benefit from a preoperative therapy rather than upfront surgical resection. Defined genomic alterations and a dynamic inflammatory microenvironment are the major culprits for disease recurrence and resistance to chemotherapeutic treatments in pancreatic cancer patients. Signal transduction pathways or tumor immune microenvironment could predict early recurrence and response to chemotherapy. In the last decade, distinct molecular subtypes of pancreatic cancer have been described, laying the bases to a tailored therapeutic approach, started firstly in the treatment of advanced disease. Patients with homologous repair deficiency, in particular with mutant germline BRCA genes, represent the first subgroup demonstrating to benefit from specific therapies. A fraction of patients with pancreatic cancer could take advantage of genome sequencing with the aim of identifying possible targetable mutations. These genomic driven strategies could be even more relevant in a potentially curative setting. In this review, we outline putative predictive markers that could help in the next future in tailoring the best therapeutic strategy for pancreatic cancer patients with a potentially curable disease.

## Introduction

Pancreatic adenocarcinoma has a dismal prognosis accounting for a 5-year overall survival (OS) rate lower than 10%. This proportion could exceed 30% when considering localized disease and surgical resection represents the only hope for cure. However, only a small proportion of patients has a resectable disease at diagnosis and local or distance relapse occurs after surgery in most of cases (1, 2).

Adjuvant therapy could prolong median disease-free survival (DFS) and OS after resection, reaching 21.6 and 54.4 months, respectively, with the most active treatment (3). Anyway, <50% of patients are disease free even after an adjuvant triplet regimen.

One of the main reasons of this aggressiveness is that even localized pancreatic cancer could be considered a systemic disease ab initio, since metastatic subclones exist before the clinical evidence of disease (4). Furthermore, circulating tumor cells could be isolated in the bloodstream of patients with pancreatic cancer, also in early stage, and their levels have been correlated with the probability of survival (5–7).

Growing evidence supports an anticipation of postoperative treatments to the neoadjuvant or perioperative setting. In fact, neoadjuvant therapy enables an early treatment of the micrometastatic disease (8). Furthermore, it improves R0 resection rate and decreases lymph node positivity rate, which translates in a positive prognostic impact (9, 10). Preoperative treatment has been also proposed as a tool for measuring *in vivo* tumor response. Moreover, after pancreatic surgery a significant fraction of patients is not fit to receive a postoperative chemotherapy due to surgical complications, poor performance status or early disease progression (3, 11, 12). Furthermore, more than 20% of patients initiating adjuvant therapy fail to complete the preplanned cycles (3). Another acknowledged advantage of preoperative therapy is the opportunity to select patients with rapid progression who would not have benefit from surgery (13).

**Figure 1:**
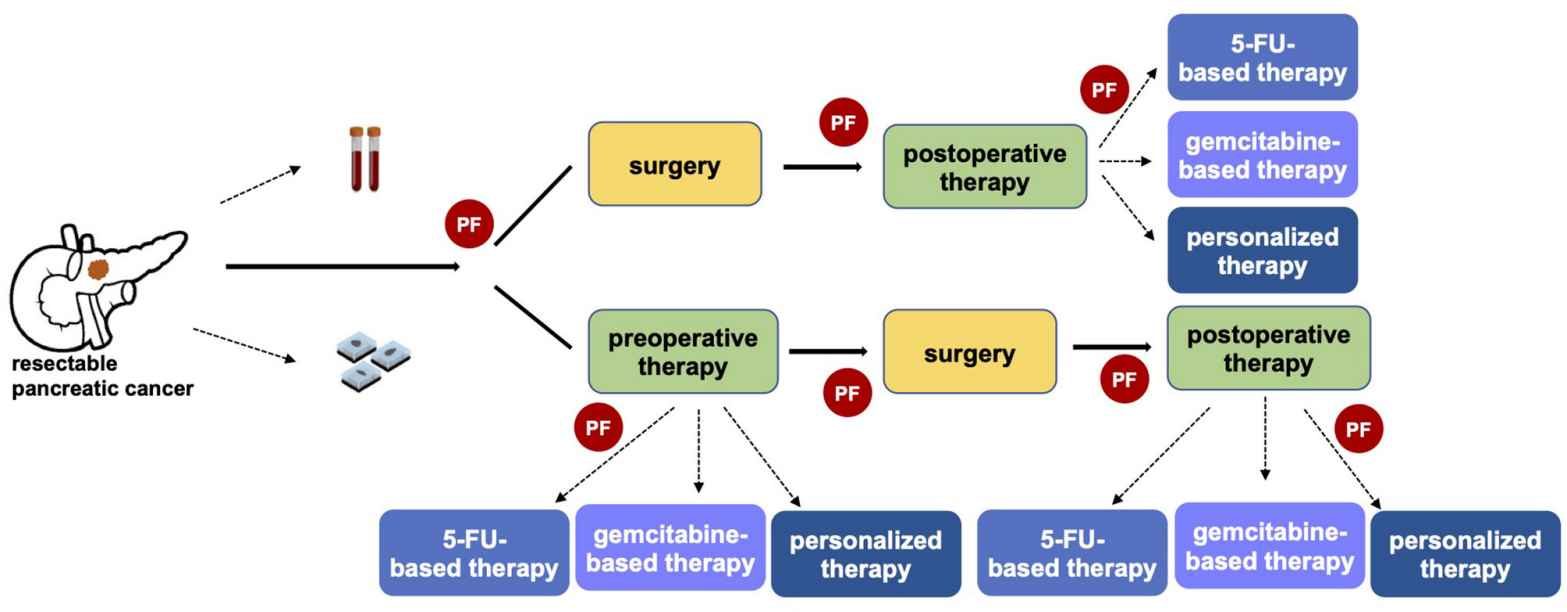
The importance of predictive factors in defining a personalized clinical approach in resectable pancreatic cancer. The identification of predictive factors (PF) has a central role in driving the therapeutic strategy, both in referring patients toward upfront surgery or preoperative therapy and in the choice of the optimal systemic pre- and post-operative therapy.

In the last decade oncological approach has been revolutionized by a growing personalization of the treatment, even if pancreatic cancer has been characterized by slower progress. To date, the decisional algorithm in localized disease is mainly based on clinical patient features and anatomical surgical criteria (14). No predictive or prognostic markers have been recognized for driving therapeutic approach in early-stage pancreatic cancer. However, a tailored strategy for selecting patients for upfront surgery vs. preoperative therapy, and possibly which type of therapy, is of utmost importance particularly in a potentially curable setting (Figure 1).

We performed a research on Pubmed/Medline, Cochrane library and Scopus using the keywords “predictive factors pancreatic cancer” OR “predictive pancreatic cancer” OR “predictive resected pancreatic cancer” OR “predictive resectable pancreatic cancer”. We selected the most relevant and pertinent studies, both in the preclinical and clinical setting, considering the quality of the studies and the relevance to the topic of this review. For ongoing clinical trials, we searched in the clinicatrials.gov database for recruiting and active phase II/III trials focused on resectable or resected pancreatic cancer.

## Adjuvant and Neoadjuvant Treatment in Resectable Pancreatic Cancer

International guidelines recommend a preoperative treatment in borderline resectable and locally advanced disease (15, 16). Whether preoperative therapy had to be offered in resectable pancreatic cancer patients remains instead matter of debate and results in this setting are controversial. The main current standard therapeutic approach of resectable disease is upfront surgery followed by adjuvant chemotherapy.

The first trial proving a significant survival increase using adjuvant therapy was the ESPAC-1 trial, in which patients with resected pancreatic cancer received adjuvant fluorouracil or only observation (17). The phase III ESPAC-3 trial did not show a statistically significant difference between adjuvant fluorouracil plus folinic acid compared with gemcitabine after resection of pancreatic ductal adenocarcinoma (18). The combination of gemcitabine and capecitabine showed longer OS compared with gemcitabine in the phase III ESPAC-4, though lack of a postoperative imaging restaging and of CA19.9 level limits for enrollment were valuable points of weakness (19). More recently, in the phase III multicenter PRODIGE 24/CCTG PA trial the modified FOLFIRINOX regimen (comprised of oxaliplatin, irinotecan, leucovorin, and fluorouracil) prolonged survival compared to gemcitabine in patients with pancreatic cancer (3). However, even with this most active regimen, more than fifty percent of patients relapse after 2 years.

The actual trend among pancreatic cancer experts in the treatment of resectable disease is to move toward neoadjuvant or perioperative treatment.

Retrospective series have firstly proposed a survival benefit of neoadjuvant therapy, especially in patients who are not fit after upfront surgery to receive adjuvant therapy (20).

The randomized phase III PREOPANC trial investigated upfront surgery with adjuvant gemcitabine compared with perioperative gemcitabine combined with preoperative radiation in patients with resectable and borderline resectable pancreatic cancer (21). Preoperative chemoradiotherapy has improved OS, together with DFS and R0 resection rate.

SWOG S1505 is a phase II non-comparative trial randomizing patients with resectable pancreatic cancer to perioperative FOLFIRINOX or gemcitabine plus nab-paclitaxel (22). Neither of the two arms met the preplanned 2-year overall survival based on historical data from adjuvant trials. Similarly, the recent phase II NEONAX trial did not meet its primary endpoint in 18-month DFS rate neither in the perioperative arm nor in the adjuvant arm with gemcitabine plus nab-paclitaxel.

Several studies investigating preoperative or perioperative therapeutic strategies are ongoing (Table 1), including the PREOPANC-2 trial (23), the nITRO trial (24), the NorPACT-1 (25), the NEPAFOX trial (26), and the PANACHE01-PRODIGE48 trial (27).

**Table 1:** Ongoing phase II/III trials investigating systemic treatment strategies in resectable pancreatic cancer.

**Trial**	**Phase**	**Condition**	**Interventions**	**Primary endpoint(s)**
**Preoperative strategy**				
NCT04141995	2	Resectable pancreatic cancer	FOLFIRINOX + digoxin	Resection rate
NCT04536077	2	Resectable pancreatic cancer	CDX-1140 +/– CDX-301	Intratumoral dendritic cells
NCT04808687	2	Resectable pancreatic cancer	Nab paclitaxel + S-1	ORR
NCT02305186	1/2	Resectable or borderline resectable pancreatic cancer	CRT + pembrolizumab	TIL infiltration; safety
NCT03750669	2	Resectable pancreatic cancer	Sequential nab-paclitaxel + gemcitabine and mFOLFIRINOX	DFS
NCT03492671	2	Resectable pancreatic cancer	Gemcitabine + nab-paclitaxel + SBRT	R0 resection rate
PRIMUS002 (NCT04176952)	2	Resectable pancreatic cancer	FOLFOX + nab-paclitaxel vs. gemcitabine + nab-paclitaxel	Time to progression
NeoOPTIMIZE (NCT04539808)	2	Resectable, borderline resectable, locally advanced pancreatic cancer	Early switching of mFOLFIRINOX or gemcitabine/nab-paclitaxel	R0 resection rate
Pancreas-CGE (NCT02818907)	–	Resectable or potentially resectable pancreatic cancer	Evaluation of survival prognostic factors	DFS
NCT04940286	2	Resectable or borderline resectable pancreatic cancer	Gemcitabine + nab-paclitaxel + durvalumab + oleclumab	
NCT03977233	2	Resectable, borderline resectable, unresectable locally advanced	FOLFIRINOX	best disease control rate by pancreatic cancer subtype
**Perioperative strategy**				
NeoPancOne (NCT04472910)	2	Resectable pancreatic cancer	Modified FOLFIRINOX	DFS according to baseline GATA6 expression level
PREOPANC-3 (NCT04927780)	3	Resectable pancreatic cancer	Perioperative vs. adjuvant mFOLFIRINOX	OS
nITRo (NCT03528785)	2	Resectable pancreatic cancer	nal-IRI + 5-FU/LV + oxaliplatin	R0 resection rate
NCT03572400	2	Resectable or borderline resectable pancreatic cancer	Neoadjuvant CCRT with gemcitabine/durvalumab + adjuvant gemcitabine/durvalumab	DFS
NCT04340141	3	Resectable pancreatic cancer	Perioperative vs. adjuvant FOLFIRINOX	OS
NCT02723331	2	Resectable or borderline resectable pancreatic cancer	Gemcitabine + nab-paclitaxel + neaodjuvant SBRT	R0 resection rate
NCT02451982	2	Resectable pancreatic cancer	CY/GVAX +/– nivolumab +/– urelumab +/– BMS-986253	IL17A expression; CD8+CD137+cells; B+PD-1+CD137+ cells; PR
**Postoperative strategy**				
NCT04969731	3	Resected pancreatic cancer	gemcitabine +/– immuncell-LC	RFS
APOLLO (NCT04858334)	2	Resected pancreatic cancer	Olaparib vs. placebo in BRCA1, BRCA2 or PALB2 Mutation	RFS
NCT04736043	–	Resected pancreatic cancer	*Ex vivo* analysis of organoid culture	OS

## Molecular Mechanisms Involved in Pancreatic Cancer Resistance to Chemotherapeutic Agents

The most promising biomarkers for supporting the benefit of a preoperative chemotherapeutic strategies in resectable PC patients emerge directly from most relevant molecular mechanisms for the intrinsic chemoresistance of pancreatic cancer cells. The resistance of solid tumors to the cytotoxic effect of cancer chemotherapy is generally accountable to the activation in key pathways involved in the regulation of cell-cycle, and, most importantly, in the suppression of programmed cell death, or apoptosis, induced by these DNA damage agents (28).

Different autocrine or paracrine pro-inflammatory factors lead to the activation of transcription factors involved in apoptosis control. Nuclear Factor κB (NF-κB) and AP-1 are among the most relevant of these transcription factors by representing a key mechanistic link between inflammation and cancer chemoresistance (29, 30). In the last decade, we contributed to demonstrate the role of the serine/threonine kinase Transforming Growth Factor-β (TGF-β)-activated kinase 1 (TAK1, also called MAP3K7) as a major determinant in the integration of different pro-inflammatory signals relevant for chemoresistance—including IL-1 (31, 32) and TGF-β (33)—to regulate, in turn, different transcription factors, including NF-κB, AP-1 (34), and YAP/TAZ (35). In this regard, TAK1 has been demonstrated as a major determinant of the resistance of cancer cells to the proapoptotic activity of chemotherapeutic agents in a number of preclinical models of solid tumors [reviewed in (36)].

More recently, with the aim of identifying circulating markers of TAK-1 pathway activation that could potentially serve as resistance biomarkers for the nanoliposomal irinotecan (nal-IRI) in patients with gemcitabine-resistant advanced pancreatic cancer, we identified CXCL8, the gene coding for IL-8, as the most significant gene regulated by TAK1 expression among those coding for secreted proteins. Consistently, circulating IL-8 was the most significant predictive marker of survival in metastatic pancreatic cancer patients treated with nal-IRI in a large panel of different cytokines, chemokines and growth factors (37). Collectively this evidence delineates a model in which autocrine or paracrine proinflammatory signaling sustain the activation of TAK-1/NF-κB cascade, and IL-8 appears to be the most significantly regulated factor by this intracellular pathway and one of the most significant candidates for selecting those patients with resectable pancreatic cancer more likely to benefit from preoperative chemotherapeutic regimens.

## Transcriptomic Classification Defines Different Molecular Subtypes of Pancreatic Cancer

Molecular subtyping could lead to many advantages, including better prognostic definition and optimizing therapeutic patient management. Gene expression profiling, mainly performed in resected tumors, outlined different subgroups, which have not yet practical applications in clinical routine. The three subtypes described by Collisson include classical, quasimenchymal and exocrine-like type (38). Bailey and colleagues identified four subtypes (immunogenic, progenitor, ADEX and squamous) (39). A two-subdivision was proposed by Moffitt, distinguishing classical and basal-like subtype (40). Although they do not overlap, the quasimenchymal (Collisson), squamous (Bailey) and basal-like subtype (Moffitt) share similar features and all three have been associated with a poor prognosis. Furthermore, these have been associated with mutations in genes involved in chromatin modification, including DNA methylation and acetylation (e.g., methylation of HNF4A and GATA6 genes). The immunogenic subtype is defined by a stromal immune infiltrate and decreased tumor cellularity).

Collisson examined untreated primary resected pancreatic cancer tumors. Quasimesenchymal subtype was characterized by high tumor grade and poor survival. The classical subtype was KRAS dependent and was correlated with GATA6 expression.

GATA6 is a transcription factor involved in the normal pancreatic development (41). High GATA6 expression has been showed to correlate with the classical phenotype, also according to Moffitt classification; viceversa, the basal-like subtype showed low GATA6 RNA expression (42). Consistent with this finding, GATA6 expression has demonstrated a prognostic value.

The negative prognostic effect of low GATA6 expression has been postulated also in resected pancreatic cancer patients (42). Furthermore, it has also been proposed that patients with basal-like GATA6^low^ tumors do not benefit from 5-fluorouracil. In an analysis of the ESPAC-3 adjuvant trial, a low GATA6 expression was correlated with lower survival in patients treated with 5-fluorouracil, while it was not associated with response to gemcitabine (43). Another group hypothesized resistance to FOLFIRINOX of the basal-like subtype (42).

The paucicellularity of pancreatic cancer makes challenging an in-depth molecular characterization. The use of patient–derived organoids has provided additional data, recapitulating the mutational landscape and transcriptional subtypes of pancreatic cancer and delineating chemotherapy signatures (44). Furthermore, they have been demonstrated to be able to predict treatment response. Interestingly, the basal-like cohort subgroup has been found to have most likely an oxaliplatin-resistant signature. Moreover, about one third of the pancreatic cancer patient–derived organoids showed no sensitivity to any of the chemotherapeutic drugs tested (gemcitabine, paclitaxel, SN-38, 5-fluorouracil, oxaliplatin). However, about half of these were sensitive to at least one of the targeted agents used.

Noteworthy, the basal-like subgroup is characterized by a hypoxia-associated gene signature, higher PD-L1 and PD-1 expression and enrichment of a T-cell-inflamed signature (42, 45).

## Role of MicroRNAs as Prognostic and Predictive Factors in Early-Stage Pancreatic Cancer

MicroRNAs (miRNAs) regulate post-transcriptional gene expression affecting physiological and pathological processes (46). Hundreds of messenger RNAs (mRNAs) may be targeted by a single miRNA (47). miRNAs are classified as oncogenic or tumor suppressor, depending on the type of activity on oncogene or tumor suppressor genes (48). A prognostic value of miRNAs has been proposed since they are involved in cell survival, proliferation, invasion and metastasis (49).

Expression of miR-574-5p, miR-1244, miR-145-star, miR-328, miR-26b-star, and miR-4321 has been associated with OS and DFS in 104 advanced pancreatic cancer patients (50).

Upregulation of miR-155, miR-196a-2, miR-203, miR-210, miR-219, and miR-222 and downregulation of miR-217 have been correlated with poor prognosis and overall survival (51). MiR-155, miR-203, and miR-222 have been related to be involved in the angiogenesis pathway (51). Furthermore, miR-155 has been linked with gemcitabine resistance in pancreatic cancer by controlling exosome synthesis (52). MiR-222, miR-203, and miR-155 are also involved in cell cycle signaling pathway (51). MiR-222 targets the cell cycle inhibitors p27 and p57, whereas miR-203 and miR-155 target p53. The tumor suppressor function of miR-217 is performed by directly targeting KRAS, since miR-217 overexpression has been correlated with reduced KRAS levels (53).

High expression of miR-200c, miR-142-5p, and miR-204 has been reported to be associated with longer survival after surgical resection of pancreatic cancer (54). Indeed, up-regulation of miR-200 has been demonstrated to reverse epithelial-to-mesenchymal transition (EMT) in gemcitabine resistant pancreatic cancer cells (55). miRNA-200 family, including 200a/200b/200c/141, and miRNA-205 regulate EMT by targeting ZEB1 and ZEB2 (SIP1), that are E-cadherin suppressing factors (56).

miR-21 is an oncogenic miRNA and regulates several oncosuppressors such as CDKN1A, PTEN, PDCD4 (57–59). miR-21 expression can modulate apoptosis, Akt phosphorylation and expression of genes involved in invasive behavior, contributing to gemcitabine resistance (60). High plasma and tissue miR-21-5p levels have been associated with worse survival in patients with resectable pancreatic cancer (60, 61). In a series of 25 resectable pancreatic cancer patients, higher preoperative levels of miR-375-3p and miR-21-5p were significantly correlated with worse OS and plasma miR-21-5p concentration was independent from other clinicopathological factors (62). Low miR-21 expression has been associated with benefit from adjuvant therapy in two different cohorts of patients with pancreatic cancer and anti-miR-21 has shown to enhance anticancer drug activity *in vitro* (63). miR-21-5p inhibits the tumor suppressor PTEN, activating the PI3K/AKT/mTOR signaling pathway (60). In fact, elevated miR-21-5p expression has been correlated with a decreased antitumor effect of gemcitabine and 5-fluorouracil (64, 65). Moreover, miR-21-5p inhibition seems to increase sensitivity to gemcitabine (66).

High presurgical levels of miR-365a-3p, which inhibits NF-κB function inducing apoptosis, and of miR-99a-5p, which regulates mTOR, are predictors of longer survival in resected pancreatic cancer patients (67). miR-221-3p has been proposed as a marker of recurrence after resection of pancreatic cancer (68). High serum and tissue levels of miR-196a-5p, which is involved in cancer proliferation and invasiveness, have been associated with inferior median OS in patients with early-stage pancreatic cancer (69). Furthermore, patients with unresectable pancreatic cancer showed higher serum miR-196a-5p levels compared to those with resectable cancer (70). Levels of plasmatic miR-182-5p demonstrated to be negative predictors of DFS and OS in pancreatic cancer (71).

Moreover, miRNA levels could help in predicting treatment response or resistance (72). Since miRNAs could affect cell cycle, drug efflux and apoptosis, they have been proposed to be involved in chemoresistance by regulating ATP-binding cassette (ABC) membrane transporters as well as exploiting intracellular effects (73).

MiR-142-5p and miR-320c were proposed as positive predictive factors of tumor response to gemcitabine (54, 74). Similarly, downregulation of mi-R-33a, mi-R-200b, mi-R-200c, mi-R-205, let-7b, let-7c, let-7d, and let-7e has been associated with gemcitabine resistance (75). Let-7 is an oncosuppressor and reduced expression of Let-7 have been correlated with cancer progression. mRNAs target of Let-7 include KRAS, HRAS, NF2, HMGA2 and LIN28 (76). The restoration of Let-7 levels in pancreatic cancer cell lines has shown to inhibit cell proliferation (77).

Furthermore, gemcitabine-resistant pancreatic cancer cells were resensitized to gemcitabine showing decreased expression of caveolin-1 and Ki-67.

A tumor suppressing role of miR-7-5p has been proposed, as its serum levels were found decreased in patients with stage III or IV pancreatic cancer compared to normal controls. Furthermore, miR-7 was shown to be significantly lower expressed in gemcitabine-resistant pancreatic cancer patients (78).

Chemoresistant pancreatic cancer cells showed P-glycoprotein overexpression associated with miR-181a-5p and miR-218-5p dysregulation (79). Plasmatic miR-181a-5p decline has been correlated with FOLFIRINOX response in pancreatic cancer patients (80). Overexpression of miR-192-5p and miR-215-5p has shown to decrease cancer cell proliferation, affecting the S-phase of cell cycle and negatively influencing response to drugs such as 5-fluorouracil (81).

In conclusion, miRNAs could play a role in guiding the best therapeutic strategy approach in early-stage pancreatic cancer patients. The tissue- and disease-specific expression and the stability in body fluids of miRNA represent their main advantages. Furthermore, they represent a potential therapeutic target based on evidence coming from *in vitro* and *in vivo* studies (82, 83).

## Molecular Profiling in Personalizing Therapeutic Strategy in Pancreatic Cancer

The molecular characterization in pancreatic cancer is not yet standard in clinical care. However, growing evidence is highlighting its relevance in the advanced setting. In a series of 71 patients whose tumor biopsies underwent whole-exome and RNA sequencing, 48% were shown to have relevant genomic alterations and 18% pathogenic or likely pathogenic germline alterations, leading to a change in clinical management in 30% of enrolled patients as a result of genomic results (84). A large US registry study showed a longer median OS in patients with actionable molecular alterations, that were about a quarter of patients screened, who received a matched therapy (85).

A recent study performing next generation sequencing (NGS) on resected pancreatic cancer specimens found likely pathogenic/pathogenic variants in 94% of samples, 18% of which were potentially actionable (86).

We recently used FoundationOne CDx or Liquid, a next-generation DNA sequencing (NGS) service to identify genomic alterations in 68 patients affected by pancreaticoduodenal cancer patients who failed standard treatments. According to ESMO Scale of Clinical Actionability for molecular Targets (ESCAT), at least one alteration ranking tier I, II, III, or IV according to ESCAT classification was detected in 8, 1, 9, and 12 patients, respectively (44.1%). Ten of them (33.3%) received a matched therapy. Patients with ESCAT tier I to IV were generally younger than the overall population (median = 54, range = 26–71 years), had an EGOG performance status score = 0 (83.3%), and an uncommon histological or clinical presentation. The most common mutations with clinical evidence of actionability (ESCAT tier I-III) involved genes of the RAF (10.3%), BRCA (5.9%) or FGFR pathways (5.9%). These results indicated that in advanced pancreaticoduodenal cancer, NGS is a feasible and valuable method for enabling precision oncology. However, this genomic profiling method might be considered only after standard treatments failure, and especially in young patients maintaining a good performance status, in order to detect potentially actionable mutations and offer molecularly targeted therapeutic approaches (87).

A clinical subgroup of patients with pancreatic cancer is identified by homologous recombination deficiency (HRD), which is caused by defects in DNA damage response (DDR) genes. The main genes that have been proposed to be involved in homologous recombination repair are BRCA1/2, PALB2, ATR, ATM, CHEK1,2, RAD51, and FANC (88). Inactivating mutations or epigenetic silencing of these genes result in HRD. The exact prevalence of HRD in pancreatic cancer has yet to be defined, partly due to the lack of a univocal definition and the variability of assays used.

If pathogenetic germline alterations have a prognostic impact remains matter of debate and available data in all stages of pancreatic cancer are conflicting (89). However, studies focusing on patients with germline mutations in DDR genes, including ATM, BRCA1/2, CDKN2A, CHEK2, ERCC4, and PALB2, have shown an improved OS (90).

Besides implications on prognosis and in terms of risk assessment and prevention, the identification of these patients has potential therapeutic repercussions (91). HRD has been identified to be a positive predictive factor of response to platinum-based therapy in patients with advanced pancreatic cancer (92–95). Furthermore, it has been speculated that the objective responsive rate of chemotherapeutic drugs containing platinum salts could reflect this subpopulation characterized by inactivation of DNA maintenance genes (96, 97). Further investigation is needed to confirm whether HRD could be a predictive factor of response to platinum therapy in early stage, since major evidence comes from advanced pancreatic cancer. However, two retrospective studies showed increased pathological complete response rates and longer OS in resectable and borderline resectable pancreatic cancer patients treated with platinum-based therapies (98, 99). Ongoing studies are investigating different therapeutic regimens in resectable and borderline resectable pancreatic cancer with HRD signature (100).

Germline BRCA mutation represents the first prospectively validated predictive factor in advanced pancreatic cancer (101). In the POLO trial the poly (ADP-ribose) polymerase (PARP) inhibitor olaparib showed to prolong PFS compared to placebo in patients whose disease had not progressed on first-line platinum-based chemotherapy [hazard ratio (HR) of 0.53, *p* = 0.004], even if it lacked to improve OS (102). Various ongoing trials are investigating the efficacy of adding PARP inhibitors in the preoperative or locally advanced setting, even in combination with radiotherapy, on the basis of a possible synergy from preclinical studies (NCT04005690) (103, 104).

Even if they represent a small proportion, mismatch repair-deficient (dMMR) tumors might benefit from immunotherapy (105, 106). The minor fraction of NTRK fusions could also represent a target for the agnostic drugs TRK inhibitors (107).

Different platforms are being used with the aim of improving precision medicine in pancreatic cancer (e.g., PRECISION-Panc in the UK, Precision Promise in the USA, EPPIC in Canada). The phase II PIONEER-Panc study is investigating novel therapeutic strategies in early-stage pancreatic cancer (NCT04481204).

Proteins involved in metabolism and mechanisms of action of chemotherapeutic drugs have been also proposed as predictive markers. Low thymidylate synthase (TYMS), excision repair cross-complementing (ERCC1) and ribonucleotide reductase M1 (RMM1) levels have been correlated with increased efficacy to fluoropirimidines, cisplatin and gemcitabine, respectively (108, 109). High levels of secreted protein acid and rich in cysteine (SPARC) have been associated with nab-paclitaxel response (110). Low expression of human equilibrative nucleoside transported 1 (hENT1) has been proposed to be involved in gemcitabine resistance (111).

Interestingly, a prospective, phase II trial enrolling patients with resectable and borderline resectable pancreatic cancer and selecting neoadjuvant therapy (fluoropyrimidine-based or gemcitabine-based) based on molecular profiling reported high resection rates (112).

## Immune Signature in Predicting Treatment Response

For several years it has been observed an increased infiltration of CD8+ and CD4+ T cells together with a reduction of FOXP3^+^ T regs after neoadjuvant therapy in pancreatic cancer patients (113, 114).

A recent study has analyzed immune blood cells in patients with pancreatic cancer after neoadjuvant FOLFIRINOX (115). Response to FOLFIRINOX was associated with increased CD8 T cell levels, in particular CD27^−^Tbet^+^ effector/effector memory subsets, while FOLFIRINOX non-responders showed higher GATA3, CCR4 and ICOS expression in CD8 T cells. FOLFIRINOX treated patients were observed to express increased Th1 cells and decreased Th2 cells, inflammatory monocytes and regulatory T cells. Similarly, in a larger series of resectable pancreatic cancer patients high Th2 cytokines of IL-4 and IP10 and low TH1 cytokines TNFα and INFγ were significantly associated with a shorter disease-free survival (116). Low γδT cell levels have been associated to worse OS in patients with borderline resectable pancreatic cancer patients treated with neoadjuvant mFOLFIRINOX (117).

Myeloid-derived suppressor cells are also involved in tumor response to chemotherapeutic treatments (118). The negative prognostic impact of high pretreatment monocyte levels in early-stage pancreatic cancer has been extensively reported (117, 119). Aiming at targeting monocytes, the combination of FOLFIRINOX with CCR2 blockade has been explored in borderline resectable and locally advanced pancreatic cancer patients in an early phase study (120). The induction of a macrophage polarization into the “classically activated” M1 phenotype by chemotherapy is still matter of debate (121, 122).

These results indicate that neoadjuvant approaches are effective not only because of their cytotoxic effect but also for the positive impact on the immune response.

## Circulating Tumor DNA as Non-invasive and Dynamic Tool

Circulating tumor DNA (ctDNA) analysis has been proposed also in pancreatic cancer as prognostic biomarker and as a tool for improving early tumor detection and monitoring tumor dynamics (123–125).

Preoperative detection of ctDNA in patients with early-stage pancreatic cancer has been correlated with decreased recurrence-free survival (RFS) and OS (126, 127). Moreover, it has been shown that a lower proportion of patients undergoing neoadjuvant chemotherapy has detectable ctDNA levels. Thus, ctDNA analysis could help in identifying patients at high risk of early recurrence after resection that might benefit from neoadjuvant chemotherapy. Some evidence has demonstrated that even high exosome DNA (exoDNA) levels are predictive of poor survival in presurgical patients (128).

Another potential application of ctDNA is the possibility of monitoring over time response to anticancer drugs. The correlation between ctDNA changes and tumor responses has been firstly demonstrated in other tumors (129–131). More recently, tumor response has been correlated with reduction of ctDNA levels also in advanced pancreatic cancer patients (132–134). Moreover, the same ctDNA dynamic trend has been observed in series of patients with resectable disease (126, 127). Therefore, serial liquid biopsies might be able to predict disease progression of on-treatment patients earlier than radiological imaging. This could be even more important in a neoadjuvant setting, where the choice of the best treatment strategy could heavily impact on patient prognosis. Furthermore, the use of liquid biopsies could find a pivotal role in patients whose tumors do not express CA19.9, that could routinely be useful in understanding clinical course.

Lastly, the mutational profile observed in ctDNA is highly overlapping compared to primary or metastatic tumor tissues (135). Thus, liquid biopsies may offer the opportunity of dynamic molecular profiling, helping in the personalization of patient treatment (136).

The most frequently used methods for measuring ctDNA levels include NGS, KRAS digital-droplet PCR assay and Safe-Sequencing System (126, 137, 138).

## Organoids Recapitulating Tumor Primitive Characteristics Could Serve for Testing Chemosensitivity

Organoids are 3D cellular structures that recapitulate the identity and the cell type diversity of the organ from which they derive. Tumor organoids are novel *ex vivo* models that can mimic the characteristic of the original tumor *in vitro*, offering an additional strategy to personalizing therapeutic approaches and are being studied in pancreatic cancer (139).

Pancreatic cancer patient–derived organoid (PDO) libraries have been generated, overcoming the challenge of low neoplastic cellularity, that characterizes pancreatic cancer. PDOs have been proposed to reflect somatic mutations of primary tumor, preserving intratumoral heterogeneity (140).

Gene expression signatures of chemosensitivity have been explored in pancreatic cancer PDOs (44). PDO profiling with DNA and RNA next-generation sequencing combined with pharmacotyping, a PDO drug-testing pipeline, may predict response to conventional chemotherapeutic agents, both in the adjuvant and metastatic setting. A PDO-derived oxaliplatin sensitivity signature has been correlated with differential response to FOLFIRINOX in patients with advanced pancreatic cancer. About one third of the pancreatic cancer PDOs were not sensitive to any of the chemotherapeutic drugs tested (oxaliplatin, SN-38, 5-fluorouracil, gemcitabine, paclitaxel) but approximately half of these showed sensitivity to one or more of the targeted agents evaluated. Pharmacotyping PDO biobank has shown a chemotherapeutic efficacy that seems to recapitulate the clinical response in patients (140).

PDO pharmacotyping has a potential role in personalizing the therapeutic approach of pancreatic cancer patients, even in the early stage. PDO cultures could facilitate molecular characterization in pancreatic cancer, since its typical low cellularity could render it difficult in primary pancreatic cancer specimens. On the other side, since their high cost, the time requested and the need of specialization, they could not yet be routinely used.

## Future Perspectives Involving Gut Microbiome

As in many other neoplasms, a significant field of research is focusing on analyzing the link between microbiota and pancreatic cancer, in which could play a role in cancer development, progression and therapeutic response (141).

The microbiota comprises trillions of microorganisms including bacteria, virus (virome), fungi (mycobiome) and archaea and is involved in host physiologic homeostasis.

Substantial abundance of microbiome in pancreatic tumors has been reported (142). Previous studies have demonstrated an association between gut microbial alteration and the presence of pancreatic cancer, but more recently a putative causative role has been also proposed (143–145).

The ability of gut microbiota to colonize pancreatic tumors can modify the tumor microbiome (146). Interestingly, flora from long survivors or healthy patients could shape tumor immune milieu, by recruiting and activating CD8+ T cells.

Moreover, the microbiome has been also proposed as predictor of postoperative survival in pancreatic cancer. Patients with resected pancreatic cancer showing a longer survival were characterized by higher tumor bacteria diversity (146). A signature including three tumor bacteria taxa *Pseudoxanthomonas, Streptomyces* and *Saccharopolyspora* and the species *Bacillus clausii* predicted patient prognosis after resection and tumor microbiome sequencing has been proposed to be used to stratify patients in adjuvant trials. Additionally, gut microbiome could impact even on postoperative complications rates after pancreatic surgery (147).

Noteworthy, recent *in vitro* and *in vivo* evidence suggests that microbiota may influence response of gastrointestinal cancers to chemotherapeutic agents. Gemcitabine metabolism has been hypothesized to be decreased by *Escherichia coli* through bacterial acetylation (148). Diverse bacteria from the class Gammaproteobacteria have been correlated with gemcitabine resistance in preclinical colon and pancreatic models (142). The upregulation of BIRC3, an inhibitor of apoptosis, caused by *Fusobacterial nucleatum* infection in colorectal cancer cells has showed decreased sensitivity to 5-FU (149). Furthermore, high levels of *Fusobacterial nucleatum* in resected colorectal cancer specimens have been correlated with 5-FU resistance and shorter DFS. It has been also suggested that in response to oxaliplatin treatment bacteria could mediate the infiltration of myeloid cells producing reactive oxygen species, which are responsible for the cytotoxic effect of oxaliplatin (150).

Fungal population is extremely represented in pancreatic cancer compared to normal pancreas and it migrates from the gut lumen to the pancreas retrogradely via the sphincter of Oddi (145). Fungi has been suggested to be involved in the process of pancreatic carcinogenesis. Recently, intratumoral mycobiome has been demonstrated to stimulate extracellular secretion of IL-33, that recruits T_H_2 cells and innate lymphoid cells 2, facilitating the type 2 immune response (151). T_H_2-polarized lymphoid cell tumor infiltration and circulating type 2 cytokines have been associated with poor prognosis in pancreatic cancer (116, 152). Interestingly, genetic deletion of IL-33 or anti-fungal treatment has been correlated with tumor burden decrease and survival prolongation in mice.

Increasing evidence is supporting a role of microbiota in pancreatic cancer progression and therapeutic response. Thus, therapeutic strategies targeting gut microbiome are under investigation and could highlight novel predictive factors and enhance treatment of pancreatic cancer.

## Conclusions

Choosing the best treatment strategy is essential in patients affected by pancreatic cancer at early stage, when a chance of cure still exists. The optimal management of resectable pancreatic cancer is debated, and a field of research is moving toward an anticipation of the standard adjuvant therapy. To date, the main factors guiding the clinical decision making include anatomical definition of resectability and clinical and biological patient aspects. However, research is focusing on the identification of novel possible prognostic and predictive markers, able to drive a tailored treatment.

In recent years, different subgroups of pancreatic cancer have been delineated based on the molecular profile. However, this has not yet found application in clinical routine.

The tumor microenvironment and immune cell infiltration have a key role in tumor progression and in resistance to chemotherapeutic treatments in pancreatic cancer patients.

A promising role of miRNAs as prognostic and predictive markers, as well as part of a potential novel therapeutic approach, in pancreatic cancer has been proposed. In the future they could contribute to drive the treatment also before or after surgery or could also become a therapeutic target. Increasing data supports the possible benefit of molecular profiling of advanced pancreatic cancer patients and some evidence is emerging in an early setting too. CtDNA could represent a tool as predictive factors of response and for monitoring the emergence of resistance in advance compared to radiological imaging. Finally, organoids, that can recapitulate typical characteristics of pancreatic cancer, could support evaluation of chemosensitivity. The growing knowledge of the association between gut microbiome and pancreatic cancer could improve the strategic and the therapeutic management of patients affected by resectable pancreatic cancer.

## Author Contributions

VM and DMe contributed to conception and design of the study. VM wrote the manuscript. All authors contributed to manuscript revision, read, and approved the submitted version.

## Funding

This work was supported by Associazione Italiana per la Ricerca sul Cancro (AIRC) through the Investigator Grant No: 23719 and 5x1000 Grant No: 12182, by the Italian Ministry of Health through the Ricerca Finalizzata 2016 GR-2016-02361134 grant and by the patient associations Nastro Viola and Voglio il Massimo through their donations to DMe.

## Conflict of Interest

The authors declare that the research was conducted in the absence of any commercial or financial relationships that could be construed as a potential conflict of interest.

## Publisher's Note

All claims expressed in this article are solely those of the authors and do not necessarily represent those of their affiliated organizations, or those of the publisher, the editors and the reviewers. Any product that may be evaluated in this article, or claim that may be made by its manufacturer, is not guaranteed or endorsed by the publisher.
